# Investigating the Daytime Visibility Requirements of Pavement Marking Considering the Influence of CCT and Illuminance of Natural Light

**DOI:** 10.3390/ijerph19053051

**Published:** 2022-03-05

**Authors:** Jiangbi Hu, Yanyan Guan, Ronghua Wang, Qingyun Cao, Yunpeng Guo, Qingxin Hu

**Affiliations:** Faculty of Architecture, Civil and Transportation Engineering, Beijing University of Technology, Beijing 100124, China; hujiangbi@bjut.edu.cn (J.H.); guanyy@emails.bjut.edu.cn (Y.G.); cqy202164129@emails.bjut.edu.cn (Q.C.); guoyunpeng@emails.bjut.edu.cn (Y.G.); hululu0716@163.com (Q.H.)

**Keywords:** daytime visibility, pavement markings, luminance contrast, detection distance

## Abstract

Pavement marking in daylight with poor quality cannot provide a reference for drivers to specify their own position relative to nearby vehicles. Luminance and Correlated color temperature (CCT) of sunlight is of importance for daytime visibility of in-service pavement markings, which lacks detailed consideration. This paper aims to explore the daytime visibility requirements of in-service pavement markings considering the influence of natural light characteristics. Based on analyzing the mechanism and impact factors of daytime visibility of pavement markings, a subjective scale of pavement markings state in the drivers’ field of view was proposed and a short and bold line was recommended as the standard state. Thirty-six tested drivers were randomly selected to detect white and yellow markings of both 15 cm and 20 cm width under 2000 to 23,000 lx and 5500 to 8500 K for outdoor natural light environment. The luminance contrast of the pavement marking to the surrounding road surface ranged from 0 to 10. The result indicated that the natural light with 2000 to 3000 lx and 7500 to 8500 K is the most unfavorable light environment for drivers to recognize pavement markings during daytime. The detection distance is becoming longer with the increase of luminance contrast. The detection distance does not increase with the increase of luminance contrast when the luminance contrast of white markings is greater than 4 and that of yellow markings is greater than 3. The model was established expressing the relationship between luminance contrast and *Q_d_* contrast. The preview time 3.65 s was selected to calculate the minimum requirements of *Q_d_* at speeds of 60, 80, 100 km/h, respectively, for different types of markings. The results can provide scientific evidence for quality evaluation and maintenance management of pavement markings in service for daytime visibility.

## 1. Introduction

Pavement marking on the road is a traffic control facility for road users to convey the rules, warnings and guidelines of road traffic to guide vehicles and pedestrians to pass safely, efficiently and conveniently. It is the core factor to ensure the stability and safety of the road traffic system [1]. Ensuring adequate round-the-clock visibility of pavement markings is the key to perform the function of the markings. The higher the visibility of pavement markings, the easier they are to be found by drivers, and the longer the detection distance provided to the drivers. Otherwise, the drivers do not have enough time to obtain and process the road outline and alignment information ahead, which is not conducive to driving safety. At the same time, when the pavement marking is not clear, it is easy to make the driver psychologically nervous, irritable and difficult to distinguish the correct lane. It is easy to generate traffic safety hazards if the driver temporarily changes lanes or misleads the vehicle behind by turning the steering wheel left and right uncertainly [2]. A study by the American Association of State Highway and Transportation Officials (AASHTO) showed that about 50% of all motor vehicle accidents in the United States are caused by cars straying from their normal lanes, resulting in a total of more than 25,000 deaths per year, accounting for almost 60% of all highway fatalities in the United States [3]. Although Montella, A. et al. studied that the frequency and severity of run-off-the-road (ROR) crashes were associated with roadway, environmental, and driver-related factors, the findings that the addition of edge markings or improved visibility of pavement markings can help drivers to clearly and accurately perceive the driving space and trajectory, reducing the risk of road lane departure and collisions had been confirmed [4]. Pavement markings are believed to provide safety benefits as they delineate the travel lanes, assisting drivers with lane positioning and providing positive guidance for roadway alignment [5].

The characteristics of daytime and nighttime light sources are different. The light environment mainly comes from the beam illuminance of the car at night on the highway, the light beam is concentrated and unidirectional. The driver almost relies on the reflective markings to provide guidance information. In contrast, during daytime, the light environment mainly comes from the sun, and the light beam is non-directional. So, the driver can identify the pavement markings through the light diffusely reflected into the eyes in all directions, and the factors affecting the visibility of the markings are more complicated. Therefore, this paper analyzes the visual mechanism of the visibility of the pavement marking for the characteristics of the daytime light environment, and investigates the factors influencing the visibility of the marking and the threshold of the marking daytime visibility evaluation index to meet the requirements of the driver’s safe visual recognition.

Asset management and maintenance report of The Conference of European Directors of Roads (CEDR) classified the marking visibility performance into daytime or street lighting visibility and nighttime visibility [6]. Daytime visibility of pavement markings can be measured using the luminance factor (*β*) or the luminance coefficient in diffuse illumination (*Q_d_*, measured in mcd·m^−2^·lx^−1^). The luminance factor and the luminance coefficient in diffuse illumination are defined in EN 1436 developed by the European Committee for Standardization, which also introduces a standard measuring geometry and classes of minimum *β* and *Q_d_* values [7]. On the basis of the standard EN 1436, the German standard ZTV M13 specifies the minimum thresholds of the coefficient of luminance (*Q_d_*) for different levels of markings in combination with the marking conditions—dry or wet, permanent or temporary, newly applied or in service [8]; the American standard MUTCD (2009) [9] and the Chinese standard GB/T 16311 (2009) [10] both specify the minimum luminance factor (*β*) to evaluate the daytime visibility of newly applied markings.

Manual on Uniform Traffic Control Devices for Streets and Highways (MUTCD) describes that changes in the color and width of longitudinal pavement markings affect the driver’s ability to perceive roadway contours [9]. Calvo-Poyo, F. et al. found that wider road markings have a speed-reducing effect by a study of the influence of the width of the longitudinal road markings on the speed of circulation [11]. Park, E. S. et al. studied the safety effect of wider edge lines by analyzing crash frequency data for road segments with and without wider edge lines, and the results showed that wider edge lines are effective in reducing crashes on rural, two-lane highways [12]. Ohme found that wider markings are more conducive to driving than 4-inch markings by evaluating drivers’ visual recognition distance of the driver’s field test [13]. Mohamed. H. et al. found that after implementing wider pavement markings, an overall reduction in both total collisions and run-off-the-road collisions by 12.3% and 19.0%, by analyzing eight years of collision and traffic data collected from 38 treatment sites (road segments) from three road authorities in Canada [14]. Similar results were reported in a before-and-after study conducted in Idaho. The study concluded that wider pavement markings can potentially reduce the frequency of total crashes by 17% and the frequency of fatal and severe injury crashes by 14%. The study also reported that wider longitudinal pavement marking could be associated with an approximately 1:25 cost-to-benefit ratio [15]. Cao, Y. analyzed the reaction time of drivers to recognize the markings through laboratory driving simulation experiments and found that white markings are more easily recognized than yellow markings [16].

The subjective perception of the drivers’ eyes on the luminance of the marking depends not only on the actual luminance value of the marking, but also on the average luminance of the surrounding road surface. Luminance contrast of the pavement marking to the surrounding road surface is an important factor affecting the driver’s reaction under different driving conditions and more suitable for measuring the visibility of markings. It represents the extent to which pavement markings stand out from the road surface background. Babić, D. et al. studied that the decrease in the contrast ratio between the marking and the road surface affects the detection quality and view range of machine-vision during daytime [17]. Under normal driving conditions, vehicles most likely maintain lane position when visibility of these markings is highest [18]. Cao, Y. [16] and Jyh-Hone Wang et al. [19] studied the effect of luminance contrast of the pavement marking to the surrounding road surface on visual recognition ability by sitting in the driver’s seat of a motionless vehicle and making responses to a series of computer-digitized video clips showing various road markings displayed on a screen in front of the vehicle. They found that the subjects’ reaction time decreased as the luminance contrast value of road markings increased. The European Cooperation in Science and Technology (COST) 331 Management Committee developed a model for the calculation of the visual information (called “visibility level”—VL) provided by road markings. VL in the COST model is related to the equivalent target size of a road marking and the luminance difference between the marking and the road surface. In the COST model, luminance is not measured directly from the driver’s point of view but rather calculated on the basis of the luminance coefficient of pavement markings and the estimated illuminance of lighting sources. The model used refers to a laboratory situation and cannot readily be applied for the complex road situation [20].

Light source chromaticity is a two-dimensional quantity, expressed in chromaticity coordinates CIE (x, y) or (u, v). However, a single-numbered metric, such as color temperature, is easier to understand and visualize than interpreting chromaticity coordinates in lighting practice. Since the beginning of the 20th century, correlated color temperature (CCT) has become one of the most widely used metrics in lighting research. Middleton et al. stated that the photometric properties of the visual scene are attenuated and scattered through the atmosphere, thus affecting the driver’s ability to recognize objects [21]. Xiao Y. et al. found that color temperature of light source and average luminance of road surface were related to the visibility of small targets using a homemade experimental system [22]. Zhang et al. found that different CCT of light source have different effects on drivers’ visual efficacy [23]. Heng T. analyzed the subjects’ recognition ability of signs in different CCT by conducting experiments and concluded that the recognition ability of low CCT was higher than that of high CCT [24].

Research on driving requirements has focused on preview times, which are calculated to obtain the required detection distance from the markings to the driver at different speeds. UK standard inspection and assessment of road markings and road studs (CS 126) describes the evaluation method of marking reflection performance for obtaining visibility distances of markings at locations where no machine survey data are available by multiplying the speed by a limited preview time of 1.8 s or a comfortable preview time of 2.2 s [25]. The limited preview time of 1.8 s is derived from the conclusion of the test reported by (cost) 331 [19], where the driver was driving at 90 km/h on a road with different detection distances of markings, and when the visual distance provided by the marking was reduced from 45 m to 30 m, the driver lost control at the curve and deviated from the original lane; so, 45 m was chosen as the limit safe detection distance of 90 km/h. The time of 1.8 s is calculated by dividing the distance by the speed as the minimum preview time in the ideal state. The CIE report 73 suggested that pavement markings should provide enough visibility distance to grant a preview time of 3 to 5 s to a motorist driving at a given speed. A true preview time of 3.0 s is recommended by the CIE report as the lower boundary. Although 3.0 s of preview time seems reasonable, the report provides no reference as to the scientific source of the recommendation [26]. Helmut T. et al. concluded that it appears necessary to allow for at least one eye fixation of 0.65 s (85th percentile duration) [27] during which the driver acquires and processes the visual pavement marking information. Therefore, they recommended that pavement markings be furnished with a minimum retroreflectance so as to provide a driver with a minimum preview time of 3.65 s (3.0 s true preview and 0.65 s for eye-fixation duration) [28]. Fan, Y. et al. applied the detection distance calculated by 3.65 s preview time to the visibility level model developed to assess the visibility of RPMs, based on drivers’ visual demands [29].

In summary, most of the current studies on the effects of marking characteristics on driving visual recognition have focused on the effects of marking width, marking color, the coefficient of luminance (*Q_d_*) or luminance factor (*β*), and the luminance contrast of the pavement marking to the surrounding road surface on daytime visibility of markings. However, except for the qualitative assessment, almost all of them are conducted in driving simulators, which have limitations as they differ from the real lighting and road environment. Existing studies have shown that illuminance and CCT, which characterize light source properties, have an effect on the drivers’ ability to recognize objects, but the studied objects are mostly small targets and signs, and there is a lack of research on the effect of daytime natural light environment properties on the visibility of pavement markings. Therefore, this paper analyzes the effect of daytime natural light environment on the visibility of markings through real daylight environment and vehicles tests to determine the most unfavorable light environment conditions during daytime. The interaction between color of markings, size of markings, luminance contrast of the pavement marking to the surrounding road surface, the coefficient of luminance (*Q_d_*) and the driving detection distance is investigated through the real vehicle test. The minimum threshold of the luminance contrast of the pavement marking to the surrounding road surface and the coefficient of luminance (*Q_d_*) under the most unfavorable daytime light environment are determined according to the driving visual requirements of markings to ensure daytime visibility requirements of markings, so as to improve driving safety and traffic efficiency.

## 2. Background

### 2.1. Analysis of the Factors Affecting Daytime Visibility of Markings

In the daylight environment with different illuminance and CCT, the daytime visibility of pavement markings is different. Drivers can perceive markings as a result of the coupling of the light source and the pavement marking as a diffuse reflector. So, in addition to the markings’ own characteristics that will affect the visibility of markings, the characteristics of the light source should also be considered. Foreign countries have tested the visual reaction time under the environment irradiated by light sources of different spectral composition. The results showed that drivers have a shorter adaptation time and good visual recognition under the environment irradiated by light sources containing short-wavelength blue-green lighting spectrum. In our research on tunnel lighting sources [30], we found that both brightness and the CCT affected the drivers’ visual recognition ability. Due to the different illuminance of the road surface and markings different time periods, the amount of light reflected into the driver’s eyes is different, and the visual stimulation felt by the driver is different. The natural light CCT and light environment luminance levels are changing at different times of the day due to the rotation of the earth and other reasons. Therefore, the driver’s perception and recognition of road markings during the day will be affected by the CCT and illuminance of natural light sources at different times from the analysis of driving safety visual recognition level.

The color, width, luminance, and luminance contrast of the pavement marking to the surrounding road surface also affect the daytime visibility of pavement markings. The reason why an object can be seen is due to the incident ray reflected from its surface into the human eyes. The more light entering the human eyes, the stronger the visual perception will be, that is, the luminance of the object depends on the amount of light reflected from the object in the direction of the observer. When two objects are placed next to each other, their luminance or chromaticity has a certain difference and the greater the difference, the easier it is for the human eyes to distinguish and recognize, otherwise it is considered the same object. During daytime, natural light beams from all directions hit the road surface and markings, which are reflected into the driver’s eyes, then the driver gets the luminance of the road surface and markings. Due to the strong illuminance of daylight during the day, the driver’s visual adaptation of the luminance level is higher than 5 cd·m^−2^, belonging to photopic and scotopic vision, when only the cone cells are active and the driver’s eyes have the form and color-vision recognition function. 

It is known that the longitudinal marking is a line with a certain width, so the greater the unit length of the longitudinal marking width, the greater the surface area of the diffuse reflection, the more the total amount of light into the driver’s eyes in the daytime natural light environment. Therefore, the width of markings should be considered for the impact of daytime visibility of markings. Chinese standards provide for different colors of markings to indicate different traffic functions, for instance, white markings for the same direction lane divider and yellow markings for the opposite lane divider. Domestic and international standards specify the different color chromaticity range of markings, which indicate that the chromaticity difference between different color markings and the road surface is different. So, the color of markings should be considered for the impact of the visibility of markings. The newly applied marking and the road surface can still be clearly visible even when they are in the shadow of a block, although they are much less bright than they would be if exposed to sunlight. This could indicate that the saliency and legibility of markings are more influenced by the relationship between the luminance of pavement markings and the surrounding road surface than by their own absolute luminance. Therefore, the luminance contrast of the pavement marking to the surrounding road surface is more applicable as an influencing factor for the visibility of markings than the luminance of markings.

### 2.2. The Subjective Scale of Pavement Marking States

Daytime sunlight beam, which diffuses to the surface of markings, is reflected by it into the driver’s eyes. When the pavement marking is considered as a secondary light source, with the increase in distance between the driver, in addition to the increased loss of propagation in the air medium, the light source irradiation area becomes larger and the unit area of energy obtained less, that is, the amount of light received by the driver’s eyes will become less. Therefore, visible markings in the driver’s field of view are rectangular surfaces made up of progressively dimming horizontal lines, and the road surfaces adjacent to the markings follow the same principle. The driver cannot identify the marking on the road surface when the luminance decreases due to the contrast of markings to road surface is not obvious. The distance between the position of the markings and the driver is the visible distance of markings. The visible distance of markings is the most intuitive characterization of the visibility of the markings. So, this paper uses the visible distance as the characterization index of daytime visibility of markings.

Based on the above analysis of the visibility of markings, we grade the status of pavement markings in the driver’s field of view in the experimental design scheme, as shown in Table 1. Considering the function of the in-service pavement markings, the state of markings in the field of view corresponding to scale 2 in Table 1 is used as the test subject’s visual recognition standard. The luminance contrast mentioned earlier is one of the influencing factors for the visibility of pavement markings. Since the luminance is influenced by various factors, such as angle and distance, the vertical luminance contrast at a height of 1.2 m is used in this study. The formula for calculating the luminance contrast is shown in Equation (1).

(1) $$C=\frac{L_{p}-L_{b}}{L_{b}}$$

where C indicates the vertical luminance contrast of the pavement marking to the surrounding road surface; *L_p_* indicates the vertical luminance of pavement marking measured in cd·m^−2^; $L_{b}$ indicates the vertical luminance of road surface measured in cd·m^−2^.

## 3. Methods

Based on the analysis of the influencing factors of daytime visibility of pavement marking, we designed a daytime visual recognition test. By collecting the visible distance of the pavement markings with different luminance contrast of the pavement marking to the surrounding road surface, different colors and different widths, and analyzing these collected data, we finally obtained the interaction between each influencing factor and the visible distance of the pavement markings, as well as the safe visual recognition requirements that the pavement markings should meet for daytime driving.

### 3.1. Samples and Scenarios

**Figure 1:**
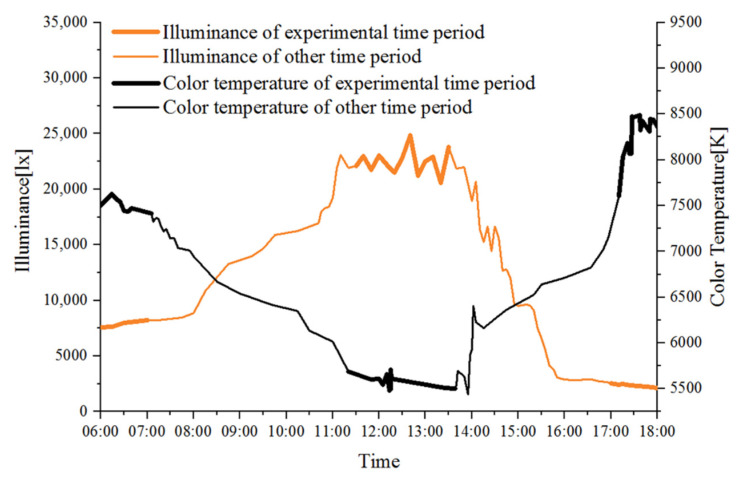
Illuminance and CCT changes from 6:00 to 18:00.

Before the formal test, we chose a clear daytime, by testing the sunlight illuminance and CCT at the test site located in Huzhou city during the time period from 6:00 to 18:00, and found the law of change, as shown in Figure 1. With the passage of time, the illuminance gradually becomes larger and the CCT gradually decreases, and remains stable when it increases or decreases to a certain value; then, the illuminance decreases and the CCT increases. Illuminance changes in the range of 2000~25,000 lx; CCT changes in the range of 5400~8500 K. Therefore, according to the above change law, three time periods with relatively large and stable differences were selected, which were morning (6:00–7:00), noon (11:00–13:00) and evening (17:00–18:00), and this time period was selected for the formal test.

As the driver’s driving position is on the left side of the lane, the right side of the field of view is more open, so the driver’s line of sight induction is easily induced by the right lane line. Moreover, the right lane line is farther away from the driver’s eyes, and the driver’s visual recognition of the right lane line is more unfavorable under the same conditions. According to the principle of the most unfavorable visual recognition, the right lane line was selected as the sample of the test visual recognition. In order to avoid the influence of weather and other conditions on the test results, the test was conducted on a 200-m long, 3.75-m wide flat and straight test section of a factory in Huzhou City, in clear weather.

**Figure 2:**
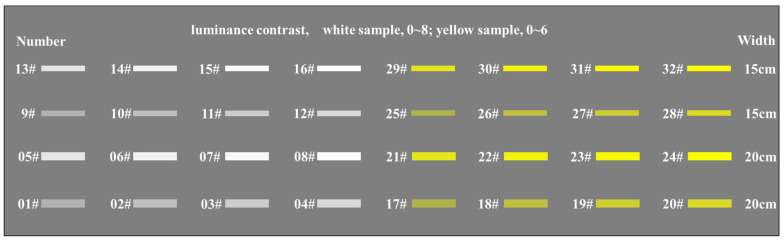
Distribution of pavement marking samples on the test road.

Chinese standard GB 5768.3 (2009) requirements for the pavement markings are white and yellow in color [31]; the Chinese standard GB/T 16311 (2009) also specifies the range of chromaticity coordinates and luminance factor *β* for white and yellow pavement markings [10]. In addition, in the operating highway, the width of 15 cm and 20 cm pavement markings are the most common. Based on the consideration of the design requirements and used types of the in-service pavement markings, the colors of the pavement marking samples used in this test were white and yellow, the width of the pavement marking is 15 cm and 20 cm, and the length is 1 m. The four types of pavement markings were applied separately on the asphalt test road, and the number of each type of pavement marking was 8. In addition, the construction process was strictly controlled so that the thickness of the 32 test pavement markings was the same and within the range of 0.4–2.5 mm. In order to make each type of pavement marking present 8 different vertical luminance contrast, heavy vehicles crush each pavement marking several times. Finally, the luminance contrast of white pavement markings to the surrounding road surfaces is in the range of 0~8; the luminance contrast of yellow pavement markings to the surrounding road surfaces is in the range of 0~6. This is shown in Figure 2.

### 3.2. Vehicle and Participants

Compared with trucks, passenger cars have the characteristics of high operating speed and low seat height, so usually, the drivers of passenger car have a narrower field of view and shorter sight distance, which is not conducive to the driver’s safety visual recognition. According to the principle of the most unfavorable visual recognition, a passenger car was selected as the test vehicle.

According to the Ministry of Public Security driver statistics in 2020, the total number of motorists in China was 456 million, of which 308 million were male drivers, accounting for 67.57%; and 148 million were female drivers, accounting for 32.43%, with a male to female ratio of about 7:3; the age of vehicle drivers is mainly between 26 and 50 years old, accounting for 71.79%. Taking into account the gender ratio and age of drivers and their visual acuity requirements, this test used the convenience sampling method to select 36 physically healthy and well-rested drivers, including 25 male drivers and 11 female drivers. Their age ranges from 20 to 50 years old (mean = 34.3 years old; standard deviation = 8.4 years old). All participants had normal and corrected-to-normal vision of 4.9 or higher, and their visual acuity tests were performed using logarithmic visual acuity charts (LVI charts). All participants passed the Yu Ziping color vision examination plates test [32] for color deficiencies and had no eye diseases such as color blindness or color weakness. The examination was performed using simple geometric figures in bright light, during which the subject was 70 cm from the surface of the figure with the line-of-sight perpendicular to the surface of the identified figure, and the subject was expected to read it within 3 s, and incorrect answers were recorded after 10 s. Answering one or more errors in the figure was judged to be color-blind or color-deficient, otherwise it was judged to be normal.

### 3.3. Experimental Instruments and Equipment

**Figure 3:**
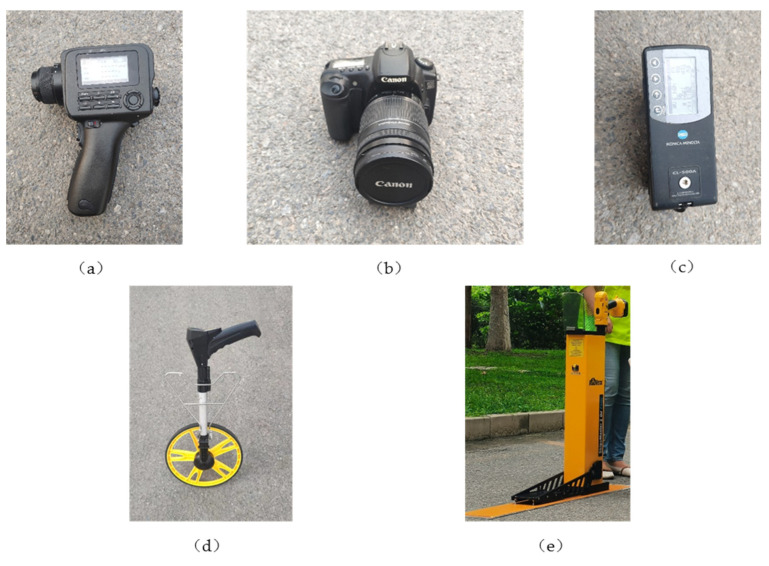
Experimental instruments and equipment. (**a**) Point luminance meter; (**b**) EOS20 camera; (**c**) Spectral irradiance meter; (**d**) Roller rangefinder; (**e**) Retroreflectometer.

Point luminance meter. A Konica Minolta CS-150 luminance meter can measure the luminance contrast of the pavement marking to the surrounding road surface, and the luminance measurement range is 0.001–299,900 cd/m^2^, with an accuracy of ±2% and repeatability of 0.2%. The CHOO calibration channel of the CS-150, which for performing measurement in accordance with the Konica Minolta calibration standard, should be selected before measuring, as shown in Figure 3a. 

Camera. The Canon EOS20 camera can take RGB mode images of the pavement marking and the background road under the same conditions to help calculate the luminance contrast. Its effective pixel number is 8.2 million, and the highest resolution is 3504 × 2336, as shown in Figure 3b.

Spectral irradiance meter. The Konica Minolta CL-500A spectroradiometer was used to measure the color rendering index, luminance, chromaticity, color temperature, and other parameters of the light source. This instrument must perform a zero calibration after it is first turned ON or after a fixed amount of time has elapsed from the last zero calibration, as shown in Figure 3c. 

Rangefinder. The roller rangefinder measures the driver’s apparent distance, i.e., the distance between the driver and the target pavement marking, as shown in Figure 3d.

Retroreflectometer. The Stripemaster 2 Touch Retroreflectometer measures the daytime luminance coefficient (*Q_d_*). The light source and sensor of the retroreflectometer for the *Q_d_* measurements meets the requirements of ASTM E2302 and EN1436. The instrument should be calibrated with a standard calibration block included with it at the beginning of the day before use, as shown in Figure 3e.

### 3.4. Experimental Procedure

**Figure 4:**
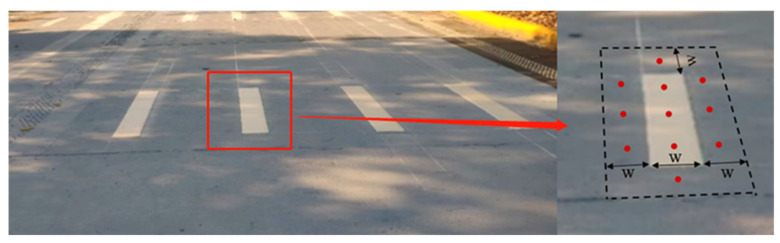
Schematic diagram of test point location.

To ensure the safety of the test and the accuracy and validity of the test data, before the start of the test, the test subjects were trained and operated two or more times to ensure that each subject was familiar with the test driving tasks before the test began. Take the 1# pavement marking sample as an example, the test steps are as follows:

1. Cover 2#~32# pavement markings with black non-reflective cloth, measure the coefficient of luminance of 1# pavement marking and its surrounding road surface and record it in the data sheet.
2. A spectral irradiance meter connected to a computer was placed on the road during the 6:00~7:00, and its probe facing the sky. The CCT and illuminance of the natural light were measured every 5 min, and at the end of each test, save its file and record the file name in the data table.
3. First, modulate the camera to output RAW file format, then use the camera to shoot the test markings vertically downwards and record the image number in the data sheet; use the point type luminance meter to measure the luminance value of 3 points of the pavement marking area and 8 points of the adjacent road surface vertically downward, as shown in Figure 4, and record it in the data sheet; the height of the camera and the point luminance meter was 1.2 m.
4. Each subject slowly drove the vehicle from a distance to approach the pavement marking area and visual recognition, and kept the vehicle in the middle of the lane, stopping the vehicle when a block that looks longer on the adjacent side and shorter on the farther side just appeared in the subject’s field of view. The distance between the driver’s seat and the middle of the marking sample was measured with a roller distance meter and recorded in the data sheet.
5. Repeat the morning measurement and visual inspection during the time period of 11:00~13:00 and 17:00~18:00.

A total of 96 sets of test data were measured for 32 pavement marking during the morning, midday and evening hours. Each set of data includes 36 drivers’ visual recognition distance, pavement marking luminance, adjacent road luminance, light environment CCT and illuminance.

## 4. Data Analysis and Results

### 4.1. Analysis of the Factors Affecting Detection Distance of Markings

**Figure 5:**
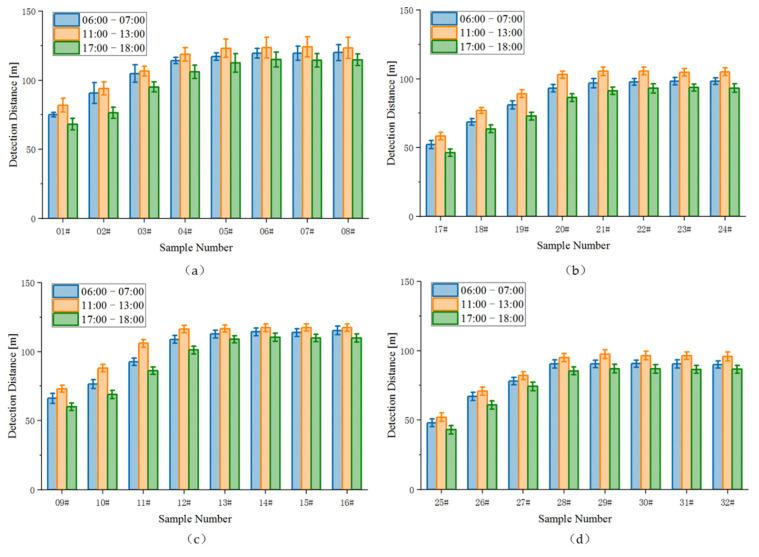
Comparative analysis of influencing factors. (**a**) White pavement markings with the width of 20 cm; (**b**) white pavement markings with the width of 15 cm; (**c**) yellow pavement markings with the width of 20 cm; (**d**) yellow pavement markings with the width of 15 cm.

The effect of natural daylight condition on visual detection distance was tested using the nonparametric test method with unknown overall distribution (the Kruskal–Wallis test was used for more than two independent groups). The finding indicates that natural daylight condition can have a significant effect on visual distance at different pavement marking samples (*p* < 0.01) but the magnitude of difference is small (Cohen’s *d* < 0.10). The average value of drivers’ detection distances is taken as the effective detection distance. Figure 5 summarizes the results for the drivers’ detection distance of pavement marking sample reported as being seen as a function of the sample number and the daytime natural light environmental conditions. The experimental pavement markings are identified in Figure 5 by luminance contrast number. See Table 2 for the daytime natural light CCT and illuminance measured at different time periods (6:00–7:00; 11:00–13:00; 17:00–18:00) in Figure 5 during the experiment. The vertical bars in the plots in Figure 5a,b represent ±1 standard deviation (*σ*). The results show that the distance of pavement marking sample visible to a driver tracks directly with the luminance contrast of pavement marking to its surrounding road surface measured under similar environmental viewing conditions. The visibility of markings is best in the 11:00–13:00 time period (illuminance in the range of 21,000~23,000 lx; CCT in the range of 5500~6500 K). While in the 17:00–18:00 time period (illuminance in the range of 2000~3000 lx; CCT in the range of 7500~8500 K), the visibility of markings is worst.

**Figure 6:**
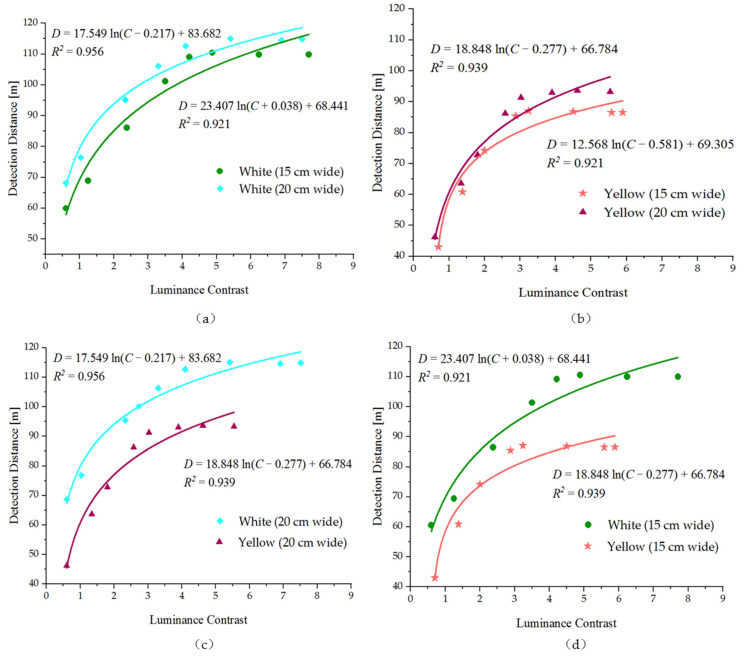
Model fitting curve of the luminance contrast of the pavement marking to the surrounding road surface and detection distance under the condition of illuminance in the range of 2000~3000 lx and CCT in the range of 7500~8500 K. (**a**) White pavement markings with widths of 15 cm and 20 cm; (**b**) yellow pavement markings with widths of 15 cm and 20 cm; (**c**) white and yellow pavement markings with the width of 20 cm; (**d**) white and yellow pavement markings with the width of 15 cm.

It is found that wider markings are more conducive to visual recognition. The values of detection distance with luminance contrast for the 20-cm wide marking is above the 15-cm wide marking, as shown in Figure 6a,b. The results show that under the same conditions of luminance contrast with the road, whether white or yellow marking, the 20-cm wide marking visibility is better than the 15-cm wide marking visibility. For the same reason, from Figure 6c,d, it can be seen that under the same luminance contrast conditions, white markings are more easily detected by drivers than yellow markings. For the same color and width of pavement marking, the distance detected by the driver increases with the increase in luminance contrast of the marking to the surrounding road, but after the luminance contrast reaches a certain value, the change in detection distance with its growth is no longer obvious.

### 4.2. Correlation between the Luminance Contrast of the Pavement Marking to the Surrounding Road Surface and the Detection Distance

Figure 6 examines more closely the relationship between the distance of pavement marking seen under the 17:00–18:00 natural light environmental conditions (illuminance in the range of 2000–3000 lx; CCT in the range of 7500–8500 K) and the luminance contrast of the marking to its surrounding road surface. The data were analyzed by using Origin statistical software, and a best-fit regression line was plotted by using the software. The results for the white 15-cm wide marking, white 20-cm wide marking, yellow 15-cm wide marking and yellow 20-cm wide marking are shown separately for reference; however, the regression line is based on the combined data. Treating luminance contrast as independent variables and detection distance as dependent variables, we employ the logarithmic function to match the relationship among the two. Equations (2)–(5) can thus be obtained, shown in Table 3. In the formulas, *C* represents the vertical luminance contrast of the marking to its surrounding road surface, *D* is the detection distance. Analysis of variance (F test) was performed on the data groups (luminance contrast and detection distance) of four kinds of markings, respectively. The analysis showed that the regression model was significant (*p* < 0.01). The goodness of fit of the test models is shown in Table 4. The analysis shows a strong correlation between the distance of marking visible and the vertical luminance contrast for the corresponding conditions, the regression model is significant with a good fitting degree.

### 4.3. Relationship between Luminance Contrast and the Coefficient of Luminance Contrast (Q_d_)

Road surfaces in the dry condition have *Q_d_* values in the range from 50 to 100 mcd·m^−2^·lx^−1^, or even higher [20]. The *Q_d_* values measured of the experimental asphalt road surface were averaged and the results were 52 mcd·m^−2^·lx^−1^. This study attempts to establish a relationship between the luminance contrast of the marking to the road surface and the *Q_d_* contrast, and we obtain the *Q_d_* safety requirement threshold of the pavement marking from the known *Q_d_* value of 52 mcd·m^−2^·lx^−1^ of the road surface. The formula for calculating the *Q_d_* contrast is shown in Equation (6).

(6) $$C_{Qd}=\frac{Q_{d-pm}-Q_{d-rs}}{Q_{d-rs}}$$

where $C_{Qd}$ indicates the $Q_{d}$ luminance contrast of the pavement marking to the surrounding road surface; $Q_{d-pm}$ indicates the $Q_{d}$ value of the pavement marking measured in mcd·m^−2^·lx^−1^; $Q_{d-rs}$ indicates the $Q_{d}$ value of the road surface measured in mcd·m^−2^·lx^−1^.

**Figure 7:**
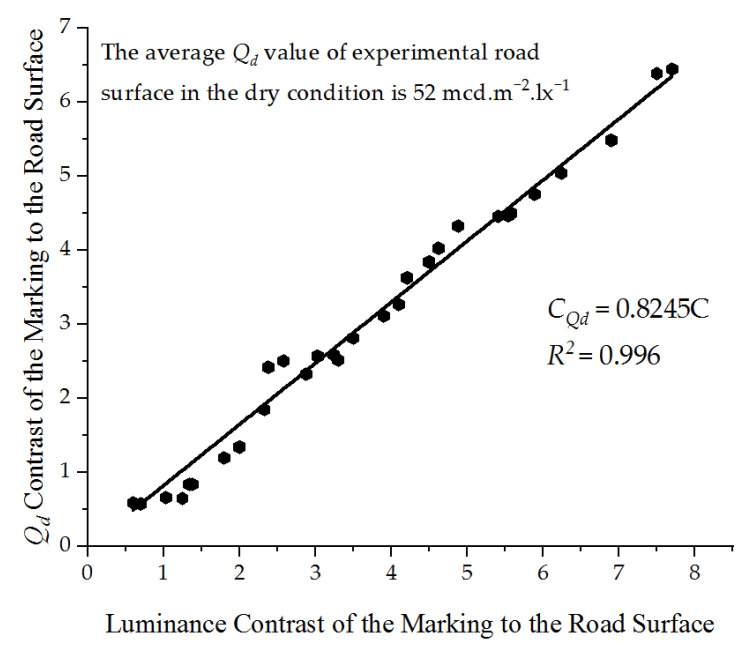
Model fitting curve of luminance contrast and the coefficient of luminance (*Q_d_*) contrast.

Figure 7 shows the relationship between luminance contrast and *Q_d_* contrast and a best-fit regression line. The final regression equation is *C_Qd_ =* 0.8245*C* (*t* = 91.778, *p* < 0.01, *R*^2^ = 0.996), which shows a strong linear correlation between the *Q_d_* contrast and the luminance contrast.

### 4.4. The Minimum Threshold for the Evaluation Index of Daytime Visibility of the Markings

The 3.65 s is selected to meet the driver’s demand for the visibility of pavement markings at different speeds. Based on the analysis of correlation between the luminance contrast of the pavement marking to the surrounding road surface and the detection distance, in this paper, the preview distance under different speeds is calculated by the preview time of 3.65 s, and the vertical luminance contrast thresholds of different types of markings under the most unfavorable daytime natural light environment at different speeds are obtained, as shown in Table 5. The data analysis of relationship between luminance contrast and the coefficient of luminance (*Q_d_*) contrast of the pavement marking to the surrounding road surface under the most unfavorable daytime natural light environment reveals that there is a significant linear relationship between the two. The *Q_d_* threshold of pavement markings to meet the requirements of driving safety visual perception is obtained by regression model calculation, as shown in Table 6, which can provide a reference for maintenance of the daytime visibility of in-service pavement markings.

## 5. Discussion

### 5.1. Daylight Environment Conditions to Meet the Demand for Daytime Visibility of Pavement Markings

The variation of daytime natural light environment had an effect on the daytime visibility of pavement markings, which was consistent for different widths and colors of the markings. The results of the three time periods of the pavement marking recognition experiment in clear and cloudless daytime shows that the detection distance is farther in the time period of high illuminance and low CCT, and closer in the time period of low illuminance and high CCT. From the CCT and illuminance trends of the daytime natural light environment, there are highest CCT and lowest illuminance at twilight. At this moment, the visibility of pavement markings is the worst in the daytime. According to the most unfavorable principle, pavement markings should meet the visibility needs of this time. There are three definitions of twilight, according to the angle of the sun reaching the horizon is divided into civil twilight, nautical twilight and astronomical twilight, and the sky luminance decreases sequentially in these three phases. However, due to the rotation and revolution of the earth, the characteristics of light sources in different cities at different latitudes and longitudes are different in different seasons. So, the twilight condition in traffic needs to be further explored.

The present study also faced limitations due to the fact that only CCT is used to express the color quality of daylight sources. CCT, simplified from a two-dimensional quantity representing chromaticity coordinates CIE (x, y) or (u, v) to a single-numbered metric that is easier to understand and visualize, is often used to represent chromaticity of white light sources. Another dimensional metric expressed in Duv, the distance to the Planck trajectory, is an important parameter for color quality of light sources for lighting in addition to the CCT. Yoshi O. found that the two numbers CCT and Duv can provide a more intuitive and accurate representation of the chromaticity coordinates of a white light source [33]. D. Durms stated that there are limitations in the application of CCT in scientific research due to the loss of information caused by reducing the spectral power distribution of a light source to a one-dimensional metric. The absolute spectral data for the daylight was not directly measured in this study [34]. Its absolute spectral power distributions of light sources with radiometric quantities and Duv are not known and should be measured in future studies.

### 5.2. The Effect Law of Luminance Contrast on the Daytime Visibility of Pavement Markings in the Most Unfavorable Daylight Light Environment

It is found that the detection distance of pavement markings increases with the increase of luminance contrast of the pavement marking to the surrounding road surface during the daytime. However, when the luminance contrast of the white pavement marking to the surrounding road surface is greater than 4 or the luminance contrast of the yellow pavement marking to the surrounding road surface is greater than 3, the detection distance of markings no longer changes with the increase of luminance contrast, which is considered to be influenced by the length of the marking. This is due to drivers’ eyes have the limit of discrimination angle, a physical quantity that describes the ability of the human eye just to distinguish between the two objects close together. As the distance of the driver from the pavement marking sample increases, the resolution angle formed by the near endpoint of the marking, the driver’s eyes and the distant endpoint gradually becomes smaller, and the length of the marking in the visual field gradually decreases, and when it is as small as the threshold, it appears as a horizontal line. With the decrease of background luminance and luminance contrast, the limiting resolution angle of the driver’s eyes increases significantly [35]. Therefore, the detection distance of pavement markings is also affected by the limiting resolution angle of the driver’s eyes when the luminance contrast of the pavement marking to the surrounding road surface is larger.

### 5.3. The Minimum Requirements for Daytime Visibility of Pavement Markings

Some factors, such as the relative position of the pavement marking recognized by the subjects to the lane (center line, edge line), the type of road where the pavement marking is located (the flat and straight road section, the road section with longitudinal gradient, the road section with horizontal curve, etc.), and the geometric elements of road alignment (longitudinal gradient, curve radius, etc.), can affect the relative position between the subjects and the marking in three-dimensional space. Accordingly, the angle of the reflected light entering the subject’s eyes will be different due to the change of spatial position information caused by the above factors. Since the pavement marking is not a perfect diffuse reflector, the change in the angle of reflection will naturally affect the amount of light reflected into the subject’s eyes and the recognition distance of the marking will change. The model parameters of contrast between markings and road surfaces and the detection distance will vary depending on the spatial information. However, whether the difference is significant needs to be verified by data from more combined scenarios. This study was conducted on a long, straight road without longitudinal gradient, and the subject’s visual recognition target was the right edge line. Therefore, the applicability of the model of contrast between markings and road surfaces and the detection distance developed in this study is somewhat limited. Nevertheless, this study has some reference significance for the study of daytime visibility requirements of markings and maintenance management of markings.

Pavement marking in daylight with poor quality cannot provide a reference for drivers to specify their own position relative to nearby vehicles. Due to the high volume of daytime traffic, pavement markings need to provide sufficient preview distance to allow drivers to react. The existing literature uses the preview time to characterize the driver’s visibility demand indicator of the marking. The preview time is the time it will take the driver to travel from the present location to the most distant road marking visible. According to the related literature, a true preview time of 3.0 s is recommended by the CIE report as the lower boundary and the preview time of 3.65 s or more proposed allows for some driving comfort. The role of comfort in driving should not be underestimated. The minimum thresholds of daytime visibility evaluation indicators of pavement markings in this paper is proposed based on a preview time of 3.65 s. Of course, the research results of this paper are still valid if other preview times are chosen to obtain the daytime visibility requirements of pavement markings.

The driver driving process can identify pavement markings and depends on the significant degree of the markings in the road background. However, the diffuse reflection performance of pavement markings and road surface in service are changing. Therefore, it seems unreasonable to set a single *Q_d_* indicator as the visibility evaluation index of pavement markings during the daytime, although some countries have provided for it in the relevant standards of pavement markings. Since the value of *Q_d_* of road surface with different materials and in service for different years are different, if a uniform *Q_d_* threshold value is used to specify the basis for pavement markings repair or reapplication, then it may result in unnecessarily high costs. Therefore, specifying the *Q_d_* thresholds of different road surface by the model of luminance contrast and *Q_d_* contrast can save economic costs and realize the refined management of in-service pavement markings maintenance.

### 5.4. The Effect of Color and Width of Pavement Markings on Its Daytime Visibility

It is found that the visibility of the white pavement markings is better than that of the yellow markings under the same conditions; the visibility of the pavement marking with a width of 20 cm is better than that of the marking with a width of 15 cm. Wider markings are better for visual recognition, which is similar to previous research in a driving simulator [11,12,13,14,15]. The driver sees the color of the pavement markings in relation to the color of the light reflected from the markings. White light is a composite light with wavelength of 400~760 nm, and yellow light is a monochromatic light of 570~600 nm. The longer the wavelength of light, the smaller the energy of individual photons. Therefore, in engineering applications, for the yellow pavement markings, by increasing its coefficient of luminance (*Q_d_*) to improve visibility is more difficult, this paper suggests that the width of the yellow markings can be increased to meet its visibility needs. For white pavement markings, a more reasonable way to improve the visibility of pavement markings can be selected by comparing the economic cost of increasing the coefficient of luminance (*Q_d_*) and increasing the width of the markings.

## 6. Conclusions

The impaired ability and accuracy of perception of vehicle position due to the poor contrast between markings and road surfaces result in an increased risk of road accidents. The influence of real daylight on daytime visibility demand of in-service markings has not been studied. For this reason, real vehicle driving tests in natural light environment and road scene are conducted in this study, and the following conclusions can be drawn from the obtained results:

1. The illuminance and CCT levels of natural light during the three time periods have an impact on the visual recognition of markings. Among them, the natural light with 2000 to 3000 lx and 7500 to 8500 K is the most unfavorable light environment for drivers to recognize road markings during daytime.
2. The visibility of the pavement marking with a width of 20 cm is better than that of the marking with a width of 15 cm. The wider markings are better for visual recognition, which is similar to previous research in a driving simulator [10,11,12,13]. The visibility of the white pavement markings is better than that of the yellow markings under the same conditions.
3. The higher the driving speed, the farther the safety sight distance required by the driver, the higher the requirement for the luminance contrast between markings and road surfaces and the *Q_d_* value of markings on the same road surface. In this study, for different types of markings, the minimum required values of luminance contrast and *Q_d_* are calculated at speeds of 60 km/h, 80 km/h and 100 km/h.

This study obtained the daytime visibility requirements of pavement marking considering the most unfavorable conditions. Therefore, further to the results of this study, we advise road authorities to properly and timely maintain the in-service pavement markings in order to increase road safety, when their visibility does not meet the visual needs of drivers.

## Figures and Tables

**Table 1:** The subjective scale of pavement marking states in the driver’s field of view.

Scales	States of Pavement Marking in the Driver’s Field of View
1	Completely disappeared
2	A short and bold line
3	A block that looks longer on the adjacent side and shorter on the farther side

**Table 2:** Illuminance and CCT of the test time period.

Experiment Time	Daytime Natural Light	
	Illuminance (lx)	CCT (K)
06:00–07:00	8000~9000	6500~7500
11:00–13:00	21,000~23,000	5500~6500
17:00–18:00	2000~3000	7500~8500

**Table 3:** Regression equation.

Equation Number	Sample Type	Daytime Natural Light		Formula	Application Range
		Illuminance (lx)	CCT (K)		
(2)	White (15 cm width)	2000~3000	7500~8500	*D* = 23.407ln(*C* + 0.038) + 68.441	*C*∈[0.5,7.5]
(3)	White (20 cm width)			*D* = 17.549ln(*C* − 0.217) + 83.682	*C*∈[0.5,7.7]
(4)	Yellow (15 cm width)			*D* = 12.568ln(*C* − 0.581) + 69.305	*C*∈[0.5,5.9]
(5)	Yellow (20 cm width)			*D* = 18.848ln(*C* − 0.277) + 66.784	*C*∈[0.5,5.6]

**Table 4:** Determination coefficient.

Equation Number	(2)	(3)	(4)	(5)
Mean square (*R*^2^)	0.956	0.921	0.921	0.939
*F* value	750.45	1794.48	686.89	940.77
*p*	<0.01	<0.01	<0.01	<0.01

**Table 5:** The luminance contrast thresholds of the pavement marking to the surrounding road surface.

Speed [km/h]	Preview Distance of 3.65 s [m]	Luminance Contrast of the Pavement Marking to the Surrounding Road Surface			
		White (15 cm Wide)	White (20 cm Wide)	Yellow (15 cm Wide)	Yellow (20 cm Wide)
60	61	0.7	0.5	1.1	1.0
80	81	1.6	1.0	2.9	2.3
100	101	4.0	2.9	---	---

**Table 6:** The coefficient of luminance (*Q_d_*) thresholds of the pavement marking.

Speed [km/h]	Preview Distance of 3.65 s [m]	Coefficient of Luminance (*Q_d_*) Thresholds of the Pavement Marking [mcd·m^−2^·lx^−1^]			
		White (15 cm Wide)	White (20 cm Wide)	Yellow (15 cm Wide)	Yellow (20 cm Wide)
60	61	82	73	99	94
80	81	121	95	176	150
100	101	223	176	---	---

## Data Availability

Data generated in this study are available upon request.
